# A Functionally Superior Second-Generation Vector Expressing an Aurora Kinase-A-Specific T-Cell Receptor for Anti-Leukaemia Adoptive Immunotherapy

**DOI:** 10.1371/journal.pone.0156896

**Published:** 2016-06-07

**Authors:** Nicholas Paul Casey, Hiroshi Fujiwara, Kazushi Tanimoto, Sachiko Okamoto, Junichi Mineno, Kiyotaka Kuzushima, Hiroshi Shiku, Masaki Yasukawa

**Affiliations:** 1 Department of Hematology, Clinical Immunology and Infectious Disease, Ehime University Graduate School of Medicine, Ehime, Japan; 2 CDM Centre, Takara Bio Inc., Shiga, Japan; 3 Division of Immunology, Aichi Cancer Center, Aichi, Japan; 4 Department of Cancer Vaccine and Immuno-Gene Therapy, Mie University Graduate School of Medicine, Mie, Japan; Institut de Génétique et Développement de Rennes, FRANCE

## Abstract

Aurora Kinase A is a cancer-associated protein normally involved in the regulation of mitosis. Being over-expressed in a range of cancers, it is a suitable target for cell-based immunotherapy. Gene transfer of T-cell receptor sequences cognisant of HLA-A*0201-restricted Aurora Kinase A antigen has previously been shown to transfer specific immunoreactivity against the target peptide in a Human Lymphocyte Antigen-restricted manner. While T cell receptor gene-transfer has great potential in overcoming the difficulties of isolating and expanding tumour-reactive lymphocytes from a patient’s own cells, one hurdle is potential mispairing and competition between exogenous and endogenous T cell receptor chains. We have used a retroviral vector design bearing a short-interfering RNA that downregulates endogenous T cell receptor chains, without affecting expression of the transgenic T cell receptor sequences. The T cell receptor expression cassette also includes a 2A self-cleaving peptide, resulting in equimolar expression of the T cell receptor alpha and beta chains, further enhancing formation of the desired T cell receptor. Via a simple, modular cloning method, we have cloned the alpha and beta chains of the anti-Aurora Kinase A-reactive T cell receptor into this ‘siTCR’ vector. We then compared the activity of this vector against the original, ‘conventional’ vector across a panel of assays. T cell receptors expressed from the siTCR-vector retained the cytotoxic functionality of the original vector, with evidence of reduced off-target reactivity. The rate of expression of correctly-formed T cell receptors was superior using the siTCR design, and this was achieved at lower vector copy numbers. Maintaining T cell receptor efficacy with a reduced vector copy number reduces the risk of genotoxicity. The siTCR design also reduces the risk of mispairing and cross-reactivity, while increasing the functional titre. Such improvements in the safety of T cell receptor gene-transfer will be crucial for clinical applications of this technology.

## Introduction

Aurora kinase A (AURKA) is a member of the serine/threonine kinase family [1, 2], and plays a role in the regulation of mitosis at the G2-M phase [2]. It is overexpressed in various cancers, including leukaemias [3, 4], and is associated with disease progression and prognosis [5, 6]. It is otherwise expressed at low levels in somatic tissues [7, 8]. The role and profile of AURKA has made it an attractive target for anti-cancer therapies, with a range of inhibitors under investigation [9, 10]. To date, however, no universal therapeutic stratagem has been identified.

Human leukocyte antigens (HLA) comprise the human major histocompatibility complex, and present candidate peptides for interrogation by the immune system. Thus target recognition by T-cell receptors is in part dependent upon the structure of the HLA, and so a given T-cell receptor is restricted to a given HLA type. Accordingly, we have previously identified an epitope of this protein which, in the context of HLA-A*0201 restriction, is able to engender a cytotoxic T cell response [4, 11]. AURKA peptides may thus be used to generate tumour-reactive CD8+ cytotoxic T lymphocytes (CTLs) [4] and CD4+/helper T cell [12] populations. While ultimately effective, the generation, isolation, and expansion of tumour-reactive lymphocytes by conventional methods is an inefficient process [13]. In order to overcome these inefficiencies, gene transfer of T cell receptors (TCRs) has proved efficient and feasible in the treatment of various malignancies [14].

However, the simple transfer of cancer-reactive TCRs to patient T cells carries its own risks that must be addressed. When targeting tumour-associated proteins, high-affinity TCRs can trigger a strong ‘on-target, off-cancer’ response to antigens occurring on normal cells [15]. Such issues necessitate careful selection of candidate TCRs, with appropriate affinities. We have previously isolated and characterised TCRs with suitable affinity for the AURKA protein, and these have proven effective *in vitro* and *in vivo*, after TCR gene transfer [11].

While CD8+ T cells, suitable for use in gene transfer therapy, may be readily isolated from patient peripheral blood [16], such cells typically bear endogenous TCRs. The addition of transgenic TCRs introduces the potential for mispairing between the α and β chains of the endogenous and transgenic TCRs. Firstly, unregulated pairing is inefficient, with up to four possible combinations, only one of which will be the tumour-reactive TCR [17–20]. These TCRs must also compete for association with CD3 molecules, which are limited in number [17–19, 21, 22]. Finally, and most importantly, the mispaired TCRs may also react against unknown, off-target antigens, with strong adverse effects [17–19, 23, 24].

A number of strategies have been tested to address these concerns. Firstly, and most simply, the formation of correctly-paired transgenic TCRs may be favoured by simply increasing the vector copy number [25]. Unfortunately this approach requires the delivery of higher vector copy numbers, with a concomitant increase in genotoxic risk [26, 27]. Secondly, a ‘lock and key’ approach, whereby the transgenic α and β chains are modified to encourage their association have also been developed, with some success [18, 23, 28].

Finally, risk of mispairing may be reduced by downregulating expression of the endogenous TCR. Zinc-Finger Nucleases have been used to delete the endogenous TCR genes [29], however these rely on triggering double-strand breaks in the genome, which again introduces a risk of genotoxicity [30, 31, 32]. The co-expression of short-interfering RNA (siRNA) with a codon-optimised transgenic TCR (a design referred to hereafter as ‘siTCR’) is another option to downregulate the endogenous TCR, without additional genotoxic risk, and without impairing expression of the introduced TCR [33, 34]. We have recently utilised this with another TCR, targeting the Wilm’s Tumour-1 protein [35], and we have commenced clinical trials with this vector (University hospital Medical Information Network Clinical Trials Registry #UMIN 0001159).

In the present study, we adapted the anti-AURKA TCR sequence into an existing siTCR retrovector backbone [33], in order to assess the efficiency of TCR formation, and cytotoxic efficacy of this vector, in comparison to a vector expressing the same TCR, but without the siTCR cassette.

We also examined the efficacy of this strategy against a model of cancer stem-like cells. Haematopoietic cells with stem-like properties are able to excrete certain molecular dyes [36], evidently through the action of membrane transporters, such as ATP-binding cassette transporter G2 [37]. This efflux activity is shared by cancer stem cells, where it is also responsible for the excretion of therapeutic drugs by these cells, resulting in a sub-population of cancer cells with stem-like properties, which have elevated resistance to drug-based cancer therapies [38]. Cells with elevated efflux activity may be identified by dye-exclusion assays, with the ‘side-population’ used as a *de facto*, but indirect, model for cancer stem cells. If cancer stem cells express Aurora Kinase A at the same elevated rate as the remainder of the cancer cell population, they should remain susceptible to TCR transfer immunotherapy.

Here we report that the anti-AURKA TCR expressed from the siTCR vector was able to bind the target peptide at a higher rate, per vector copy number, than the conventional vector design. The siTCR design also retained the sensitivity and efficacy of the original TCR design, and was effective against AURKA-positive acute myeloid leukaemia (AML) cells, including side-population cells.

## Materials and Methods

Approval for this study was obtained from the Institutional Review Board of Ehime University Hospital. Written informed consent was obtained from all patients and healthy volunteers in accordance with the Declaration of Helsinki.

### Cell lines and PBMCs

C1R-A2, a HLA-A*0201+ Epstein-Barr virus-immortalized B-lymphoblastoid cell line [39], HLA-A*0201+ AML cell lines GANMO-1 [11] and OCI-AML3 [40], as well as the HLA-A*0201+ Adult T-cell Leukaemia (ATL) cell line, TL-Om1 [41] were cultured in RPMI with 10% foetal calf serum (FCS) (Gibco), and penicillin and streptomycin. 293T and PG-13 cells (both from the American Type Culture Collection, catalogue numbers CRL-3216 and CRL-10686, respectively) were cultured in DMEM (Gibco) with 10% FCS, and penicillin and streptomycin (Gibco).

Peripheral blood monocytes were isolated from whole blood of healthy donors by Ficoll-Conway separation using standard protocols. CD8+ CTLs were subsequently enriched by magnetic bead selection (Human CD8 MicroBeads, Miltenyi Biotech). Cells were then either used fresh, or stored at -80°C in ‘Cellmenity’ solution (Waken Tech).

### Vector Construction and Virion Production

Genes for HLA-A*0201-resticted, AURKA_207-215_-specific TCR-α and TCR-β chains [11] were cloned with an InFusion kit (Takara Bio) into a pSplice-a2Ab-siTCR vector [33] to generate ‘siAUK’ vectors (S1 Fig). The constant regions of the α- and β-chains were codon optimised to escape interference by the siTCR expressed by the pSplice-a2Ab-siTCR vector. An existing vector bearing the anti-AURKA α- and β-chains (Takara Bio) [11] was used as a control (‘coAUK’).

Ecotropic, VSV-G pseudotyped retrovectors were generated by transient co-transfection of HEK-293T cells by Calcium-Phosphate precipitation with a Retrovirus Packaging Kit (Takara Bio). This supernatant was filtered, and used to transfect PG13 packaging cells to generate GaLV-pseudotyped retrovirus particles, as described previously [4].

### Transduction of CD8+ Cells

Freshly isolated, or thawed and washed, CD8+ cells were stimulated on anti-CD3-coated plates (prepared in advance with 1μg/ml of OKT3 LEAF-purified monoclonal antibody (BioLegend) in PBS). Cells were cultured in GT-T503 medium supplemented with 5% human serum, 0.2% human serum albumin, 50U/ml IL-2, 5ng/ml IL-7, 10ng/ml IL-15, 10ng/ml IL-21, with penicillin and streptomycin.

Retroviral supernatant was pre-applied to plates coated with RetroNectin (Takara Bio), and cells were transduced on days 2 and 3, as described previously [34].

On day 5, where appropriate, cells expressing the transgenic, Variable β chain 12 (Vβ12) TCR were bead-enriched, using FITC-conjugated anti-Vβ12 antibodies (Beckman Coulter), anti-FITC magnetic beads (Miltenyi Biotech), and MACS separation columns (Miltenyi Biotech), as per the manufacturer’s recommendations.

### Flow-Cytometry

For flow-cytometric analysis, approximately 5x10^5^ cells were washed in PBS with 0.1% FCS. Where appropriate, cells were first labelled with PE-conjugated AUK_207-215_/HLA-A*0201 tetramer. Cells were washed twice more before double-staining with VB12-FITC and APC-conjugated anti-CD8 antibody (BD Pharmingen). Cells were washed thrice more, 7AAD was added (Life Technologies), then cells were analysed on a Gallios machine and software.

### qPCR

#### Proviral copy number

Genomic DNA was collected from transduced cells on day 5/day of FCM analysis with a QIAmp Blood Mini Kit (Qiagen). qPCR was performed with SensiFAST Probe Lo-Rox reagent (BioLine), on an ABI Prism 7500 Sequence Detection System (Applied Biosystems/Life Technologies), using the following primers;

Primers for Interferon-gamma housekeeping gene-

IFNgFP; GACTTGAATGTCCAACGCAA

IFNgRP; TTACTGGGATGCTCTTCGAC

Primers for MLV LTR-

MLVFP; GGGTACCCGTGTATCCAATA

MLVRP; TGACGGGTAGTCAATCACTC

#### AURKA mRNA assay

RNA was collected from target cells using a Qiagen RNeasy kit (Qiagen), then reverse transcribed to cDNA with MultiScribe Reverse Transcriptase (Applied Biosystems/Life Technologies). AURKA Taqman probe Hs00269212_m1 (Applied Biosciences), and GapDH 4326317E (Applied Biosciences) as an internal control, were used with Universal PCR Master Mix (Life Technologies) on an ABI Prism 7500 Sequence Detection System (Applied Biosystems/Life Technologies).

### Chromium-release Assays

To assess the cytotoxicity of the different effector cell types, 5x10^3^ Cr^51^-labelled target cells were co-cultured with effector cells as per standard protocols in 200μl RPMI with 10% FCS, in 96-well round-bottom plates. Peptide-pulsing was carried out with the indicated concentrations of AUK_207-215_ peptide (Thermo Electron) for 2 hours at 37°C before co-culture with effector cells, at a range of effector:target (E:T) ratios, as indicated. To assess HLA-restriction, target cells were incubated with 10μg/ml anti-human HLA-A,B,C (Biolegend #311412) or anti-human HLA-DR (Biolegend #307612), for 60 minutes at 37°C.

After 4 hours co-culture, 100μl supernatant was collected from each well, and assayed with an AccuFLEXγ 7000 scintillation counter.

### CD107a Degranulation Assays

CD107a (or ‘lysosome-associated membrane protein 1’) labelling was used to provide a measurement of lysosome release by T cells, providing a quantitative measure of response to stimulation by target cells. To assess CD107a expression by each effector cell type, Mitomycin-C (MMC) treated target cells and effector cells were co-cultured in the presence of 3μl (APC) fluorophore-labelled CD107a (Mouse anti-human CD107a-APC, BD Pharmingen), prior to flow-cytometric analysis with fluorophore-conjugated labelled anti-CD8 and anti-Vβ12, as described previously [11].

### ELISA Assays

In preparation for ELISA-based analysis of Interferon Ɣ (IFNγ), Tumour Necrosis Factor α (TNFα), and Interleukin-2 (IL-2) expression, effector:target co-cultures were prepared in 2:1 ratios, under the conditions described, in 200μl GT-T503 supplemented with 5% human serum, in 96-well, round-bottomed plates. Cells were co-cultured for 24 hours, before supernatant was collected and assayed fresh, or stored at -80°C until use.

### Isolation and Characterisation of ‘Side Population’ cells

For side-population (SP) experiments, GANMO-1 cells were labelled with 5μg Hoechst 33342 (Sigma) per 10^6^ cells, per ml of RPMI with 10% FCS, for 90 minutes at 37°C. Cells were washed once, and resuspended in 4ml RPMI with 25% FCS, then labelled with Propidium Iodide (Sigma) at 5μg/ml, on ice for 30 minutes. Dye-effluxing ‘side-population’ cells were considered to be those low in both Hoechst red and blue channels. Cells were sterile-sorted by fluorescence-activated cell sorting (FACS), into RPMI with 25% FCS. For sorted control cells, an arbitrary ‘non-side population’ (NSP) population was gated to represent the remaining cells. Variations from this protocol are indicated where appropriate.

Dye-efflux by SP cells derives from elevated activity of ABC transporters. Therefore, characterisation of the SP cells was carried out by inhibiting these transporters with Verapamil prior to Hoechst 33342 labelling [42]. Briefly, GANMO-1 cells were pre-incubated with 500μM Verapamil for 15 minutes at 37°C, prior to Hoechst labelling and flow cytometric analysis.

Hoechst 33342 at certain doses can be toxic in some cell types [43, 44]. We found that the 5μg dose impaired viability of GANMO-1 cells beyond approximately 12 hours, however they were tolerant of lower doses (data not shown). Therefore, [43, 44], for experiments requiring culture beyond 24 hours, GANMO-1 cells were labelled with 1μg/ml/10^6^ of Hoechst 33342, prior to sorting. The dye-effluxing side-population remained distinct under these conditions.

To characterise the progenitive capacity of the SP cells, GANMO-1 cells were labelled with Hoechst 33342 at 1μg/ml/10^6^ cells, and sorted by FACS. These cells were used in the following two extended culture assays.

In order to assess the ability of SP cells to give rise to both SP cells and non-SP cells, sorted cells were cultured in RPMI with 20% FCS, in round-bottomed, 96-well plates. After 12 days, they were labelled with Hoechst 33342 at 5μg/ml/10^6^ cells, and analysed by flow cytometry.

To assess the proliferative potential of SP cells, sorted cells were also cultured in soft agar, using a method based on Cifone and Fidler (1980) [45]. Briefly, 50μl of 0.6% agar in RPMI with 20% FCS was set in a 96-well, flat-bottomed plate. Sorted cells were mixed to a final suspension of 0.4% agar with 20% RPMI, and 75μl of this suspension was added per well, for approximately 1000 cells/well. After setting, 100 μl of RPMI with 20% FCS was added on top. Representative non-SP cells were also sorted, and used as a control. This experiment was performed in triplicate. After 12 days, discrete populations of cells were counted. A ‘seed location’ was defined as a cell or grouping of cells that was physically discrete from other cells, and therefore likely derived from a single mother cell. Locations from each of three samples were assessed, and the number of cells in each location counted. Finally, for surface marker characterisation of SP cells, GANMO-1 cells labelled with Hoechst 33342 at 5μg/ml/10^6^, then also labelled with CD33-APC (BD Pharmingen), c-Kit-FITC (CD117-FITC, eBioscience) and CD38-PE (Beckman Coulter), before analysis by flow cytometry.

### Statistical Analyses

Correlation coefficients were compared using Preacher’s calculation for the test of the difference between two independent correlation coefficients (www.quantpsy.com). Pairwise t-tests and one-way ANOVAs were performed within Microsoft Excel. Tukey’s post-hoc analyses were performed with the online analysis tools at http://statistica.mooo.com.

## Results

### Superior expression of AURKA-specific TCR molecules from the siTCR vector design

To compare rates of formation of AURKA-cognisant TCRs, CD8+ cells were transduced with either the siAUK vector, or the conventional, control vector (coAUK), at a range of multiplicities of infection (MOIs). Where appropriate, non-gene modified cells (‘NGM’) were included as controls. Binding of PE-conjugated AUK_207-215_ tetramers, and expression of Vβ12 were assessed by flow cytometry. The number of integrated vector copies (Vector Copy Number, or ‘VCN’), was determined by qPCR of gDNA samples [25].

**Fig 1:**
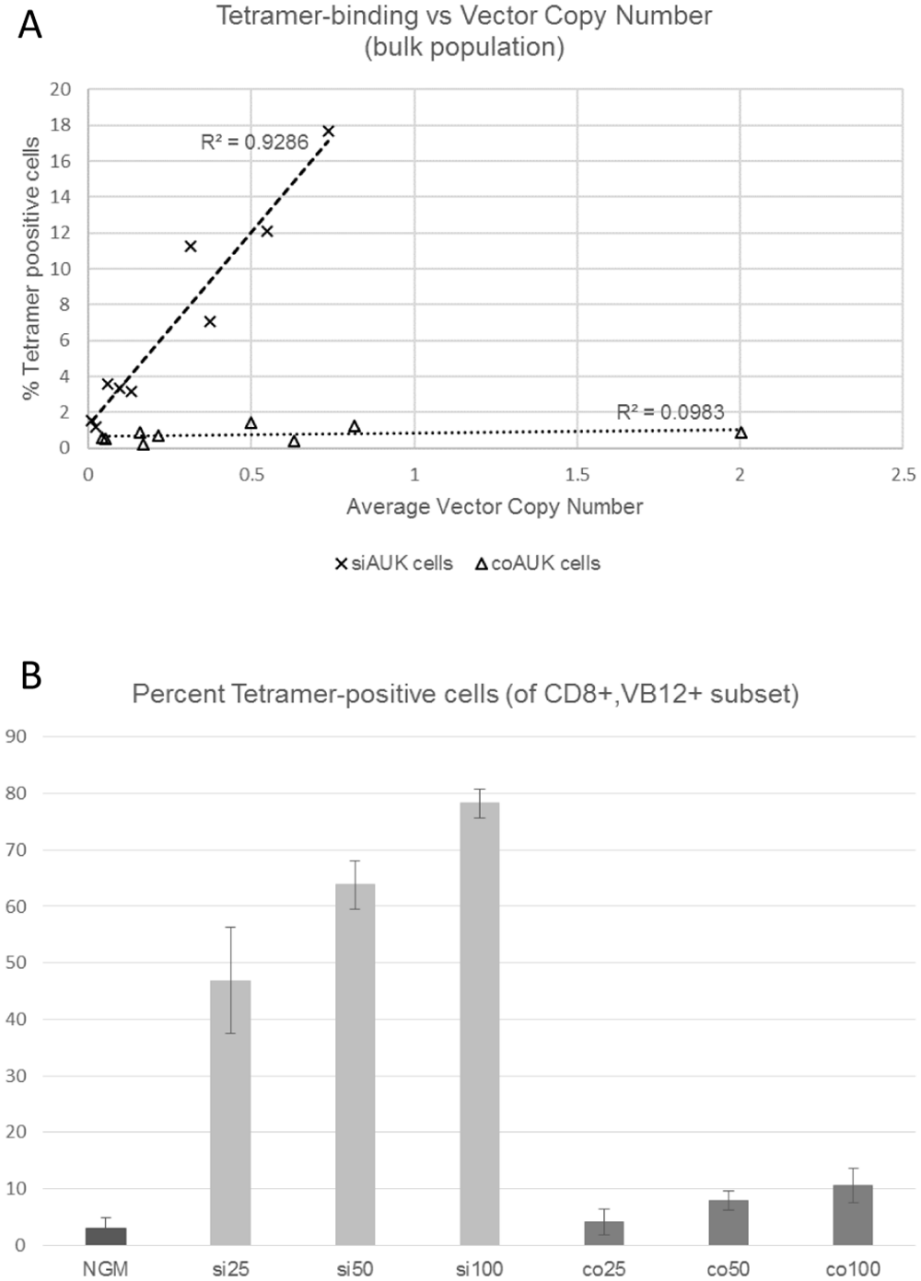
Binding of AURKA tetramer by transduced CD8+ effector cell populations. CD8+ cells from three donors were transduced with siAUK or coAUK vectors, at a range of MOIs (vector supernatant applied to Retronectin-coated plates at 25%, 50%, and 100% of usual volume, diluted where appropriate with fresh culture medium). Binding of fluorophore-conjugated AUK_207-215_/HLA-A*0201 tetramer was analysed by flow cytometry. Genomic DNA was also collected, and vector copy number determined by qPCR. (A) Tetramer-binding and vector copy number were compared for the population as a whole. Correlation coefficients were compared using Preacher’s calculation (p<0.05). (B) Cells were also labelled for CD8+, and Vβ12 transgene expression. Cells from each MOI sample were gated for CD8 and Vβ12 positivity, and the percentage of tetramer-binding cells was recorded.

With both vectors, we observed a near-linear relationship between average vector copy number and tetramer-binding across the range of MOIs used (Fig 1A), however the average rates of tetramer-binding per VCN for the siAUK cells was superior to that from the conventional vector. Rates of Vβ12 transgene expression per VCN were similar for both vectors (S2 Fig, p>0.05), whereas tetramer-binding as a function of Vβ12 expression was also superior for the siAUK vector (S3 Fig, p<0.05). These results indicate that the superior rate of tetramer binding was a function of vector design, and not differential transgene expression. Finally, we found that the percentage of tetramer-binding cells, amongst transduced cells, was higher in the siAUK sample compared with the coAUK sample (0.0 1B).

### Superior Sensitivity, Specificity and Efficacy of siAUK TCR

To confirm that the siAUK vector design retained the cytotoxic efficacy of the coAUK vector, Vβ12-enriched effector cells were co-cultured at E:T = 2:1 with HLA-A0201^+^ cells that had been loaded with AURKA peptide at 10-fold dilutions (from 1μM to 0.001μM). Responsiveness of effector cells was assessed across a range of assays.

**Fig 2:**
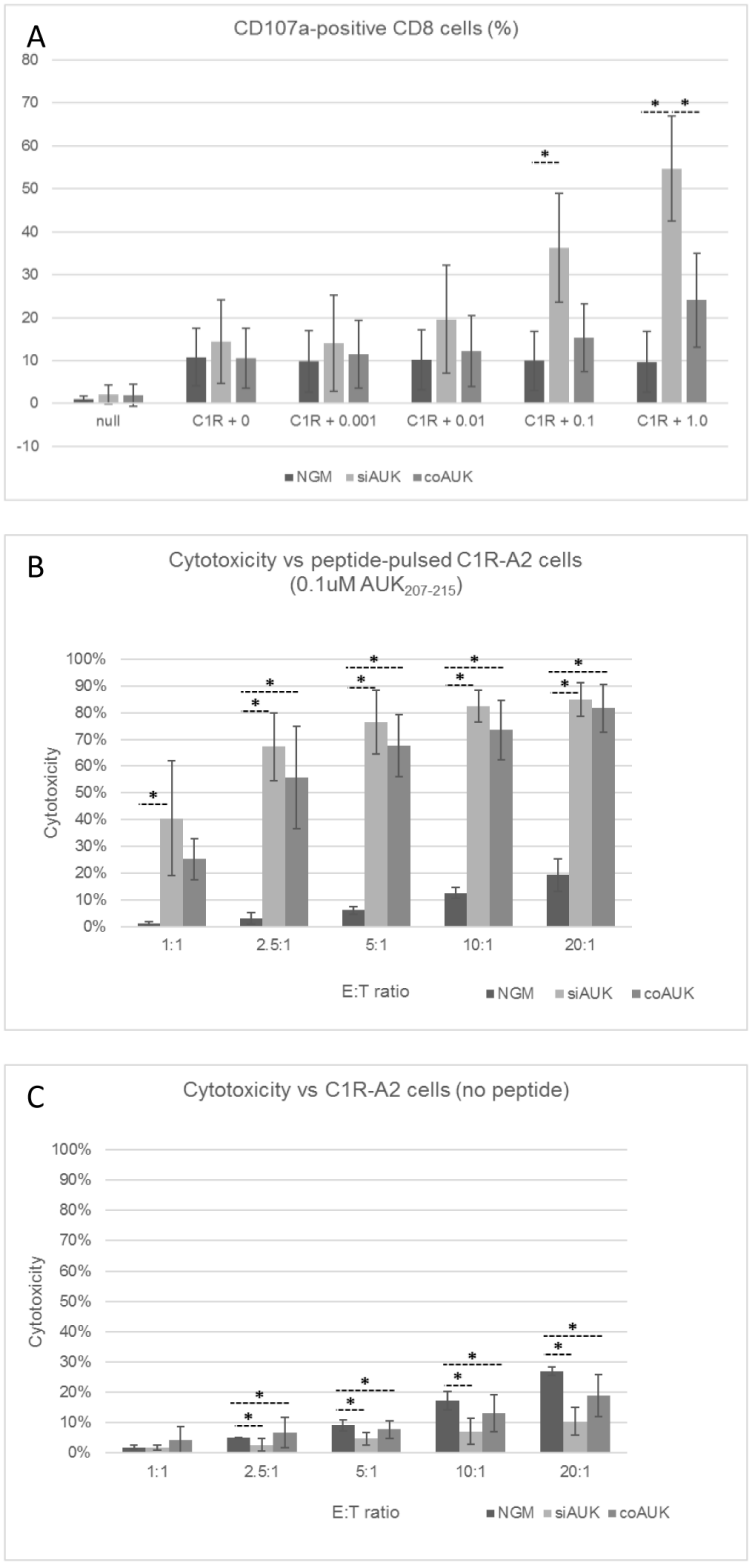
Sensitivity and Cytotoxicity by CD8+ cells in response to peptide-loaded target cells. CD8+ cells were transduced with siAUK or coAUK vectors, and enriched on the basis of Vβ12 expression. NGM cells were used as a negative control. (A) To measure CD107a degranulation, C1R-A2 cells were pulsed with the indicated concentration of AUK_207-215_ peptide. Effector cells were co-cultured with target cells at E:T = 2:1. CD107a-APC antibody was added prior to 3 hours co-culture. CD107a labelling was assessed by FCM, and is presented as a percentage of CD8+ cells. For Chromium-release assessment of total cell killing, TCR-enriched T cells were co-cultured at a range of E:T ratios for 4 hours with Cr^51^-labelled, C1R-A2 target cells that were pulsed (B) or not pulsed (C) with 0.1μM AUK_207-215_ (* p<0.05, One-way ANOVA with Tukey’s test, n = 3, ± S.D.).

When comparing the CD107a degranulation response, at lower peptide concentrations, the percentage of siAUK cells that were CD107a-positive was not significantly different to the percentage of coAUK cells (Fig 2A). At the highest peptide concentration, however, the percentage of CD107a-positive siAUK cells was significantly greater than coAUK cells (1μM, p<0.05, One-way ANOVA with Tukey’s test, n = 3).

For the total cell killing (Chromium-release) assays, at most E:T ratios, the total cell killing response was comparable between siAUK and coAUK cells (Fig 2B).

In the absence of AUK_207-215_ peptide, NGM cells still exhibited cell killing that increased with E:T ratio (Fig 2C). By contrast, total cell killing by siAUK cells in the absence of AUK_207-215_ peptide was significantly lower than by NGM cells at all E:T ratios from 2.5:1 upwards. Total cell killing by coAUK cells was also significantly lower than by NGM at E:T ratios of 5:1 and above.

### Reactivity to Cells with Endogenous AURKA Expression

In order to confirm cytotoxicity against leukaemic cells with endogenous expression of AURKA, HLA A*0201+ AML cell lines with elevated levels of AURKA mRNA expression (S4 Fig), were co-cultured with the effector cells.

**Fig 3:**
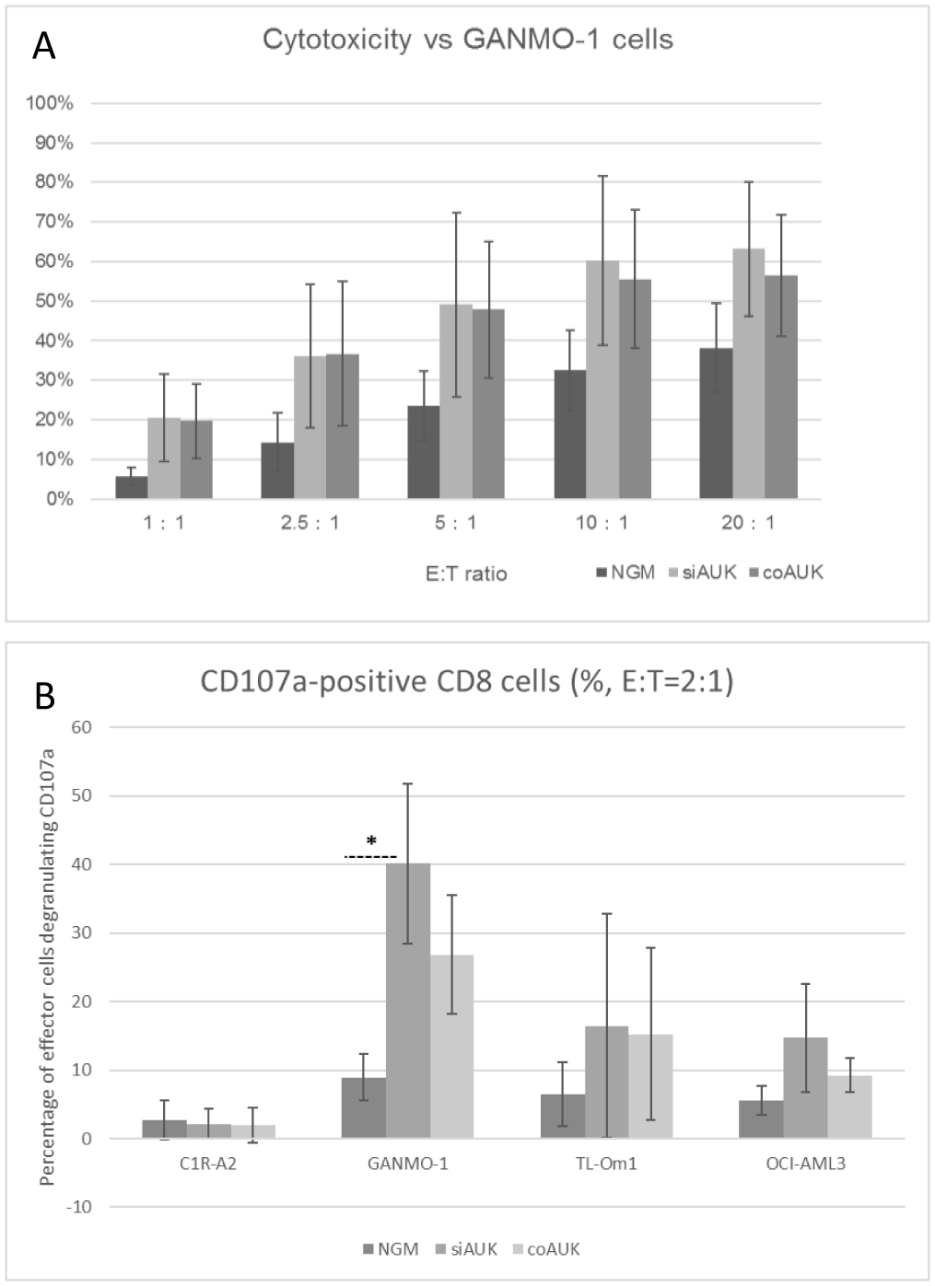
Cytotoxic response against AURKA-positive GANMO-1 cells. (A) Total cell killing of Chromium-labelled GANMO-1 cells was assessed by Chromium release after 3 hours co-culture with NGM, coAUK, or siAUK effector cells. (B) Effector cells were co-cultured (E:T = 2:1) for 3 hours with a range of HLA A*0201+ target cells in the presence of CD107a antibody (C1R-A2 is an AURKA-negative control). CD107a degranulation was assessed by flow cytometry (* p<0.05, One-way ANOVA with Tukey’s test, n = 3, ± S.D.).

Total cell killing by the two transduced effector cell populations (Fig 3A) was similar at each E:T ratio. CD107a degranulation by effector cells was also examined (Fig 3B). Here the response by the siAUK effector cells for each target cell type was at least as high as the response by coAUK cells, and the response to GANMO-1 cells was significantly higher than the response by NGM cells.

### Persistence and Expandability of Gene-modified CD8 cells

The potential of the siAUK and coAUK effector cell populations to survive and expand was assessed by extended culture *in vitro*, with weekly stimulation at E:T = 2:1 with peptide-loaded (0.1μM), MMC-treated C1R-0201 cells. In order to test the persistence and expansion of transduced cells within a mixed population of effector cells, we used non-enriched cells. Proliferation of effector cells was determined by weekly cell counts. The rate of tetramer-binding, and CD107a degranulation in response to stimulation, were assessed by flow cytometry.

**Fig 4:**
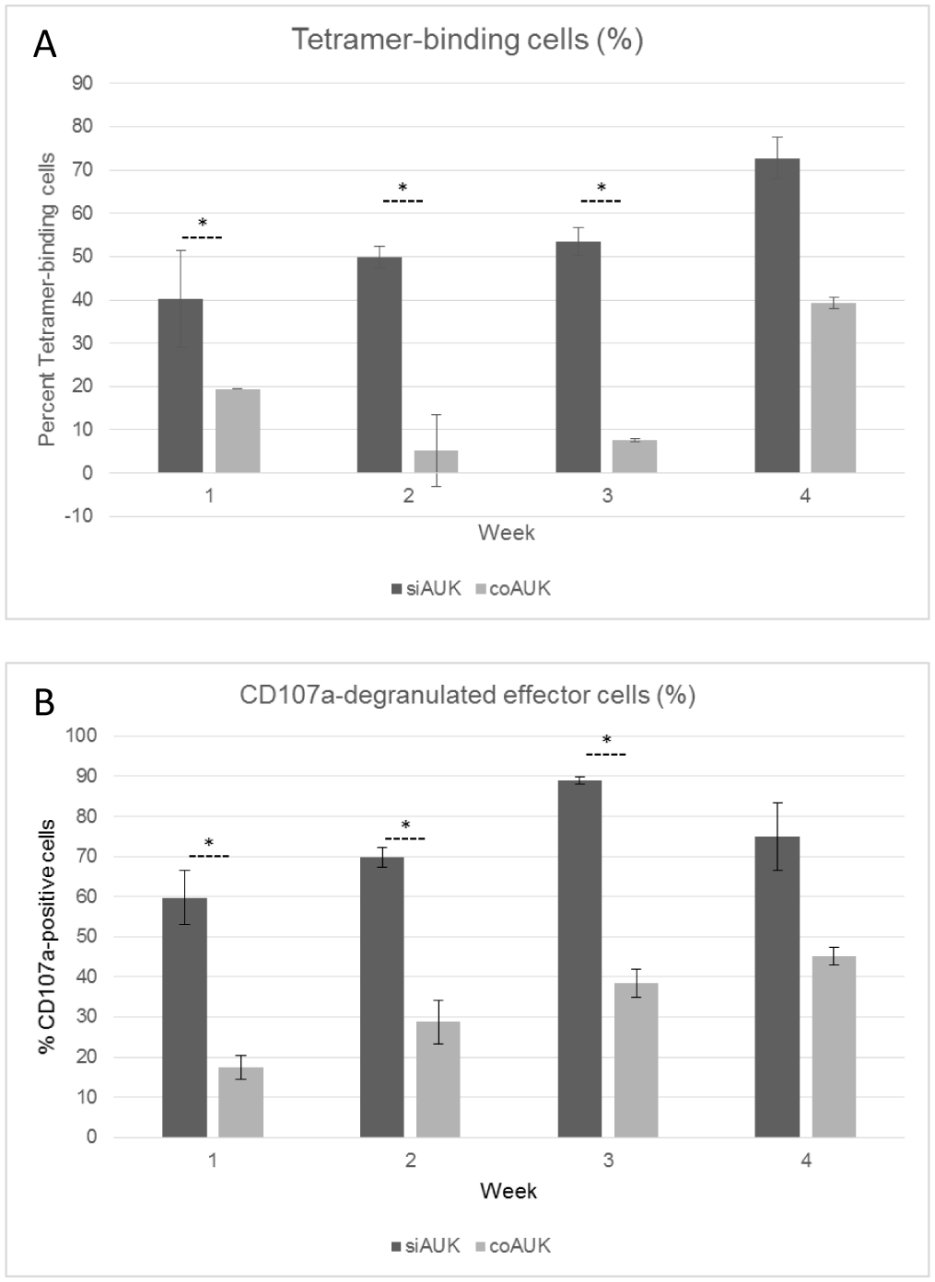
Enhanced persistence and expandability of siAUK-transduced CTLs. Bulk siAUK or coAUK cells (ie. non-enriched) were stimulated weekly with peptide-pulsed C1R-0201 cells at E:T = 2:1. (A) Tetramer-binding was assessed by labelling with a fluorophore-conjugated AUK^207-215^ tetramer, and expressed as a percentage of Vβ12+ CD8 cells. (B) CD107a degranulation was assessed in response to stimulation, and the number of CD107a-positive cells was expressed as a percentage of Vβ12+ CD8 cells.

As per our other results, the percentage of tetramer-binding cells was higher amongst siAUK population at the commencement of the study (Fig 4A). This superiority persisted, because while the percentage of tetramer-binding cells in both populations increased in response to repeated stimulation, the percentage within the siAUK population was significantly higher for the first three weeks (Fig 4A). Furthermore, of the Vβ12-positive cells in the siAUK and coAUK populations, the percentage of cells exhibiting CD107a degranulation in response to stimulation was also significantly higher amongst the siAUK cells for the first three weeks (Fig 4B).

### Acute Myeloid Leukaemia ‘Side Population’ Cells

**Fig 5:**
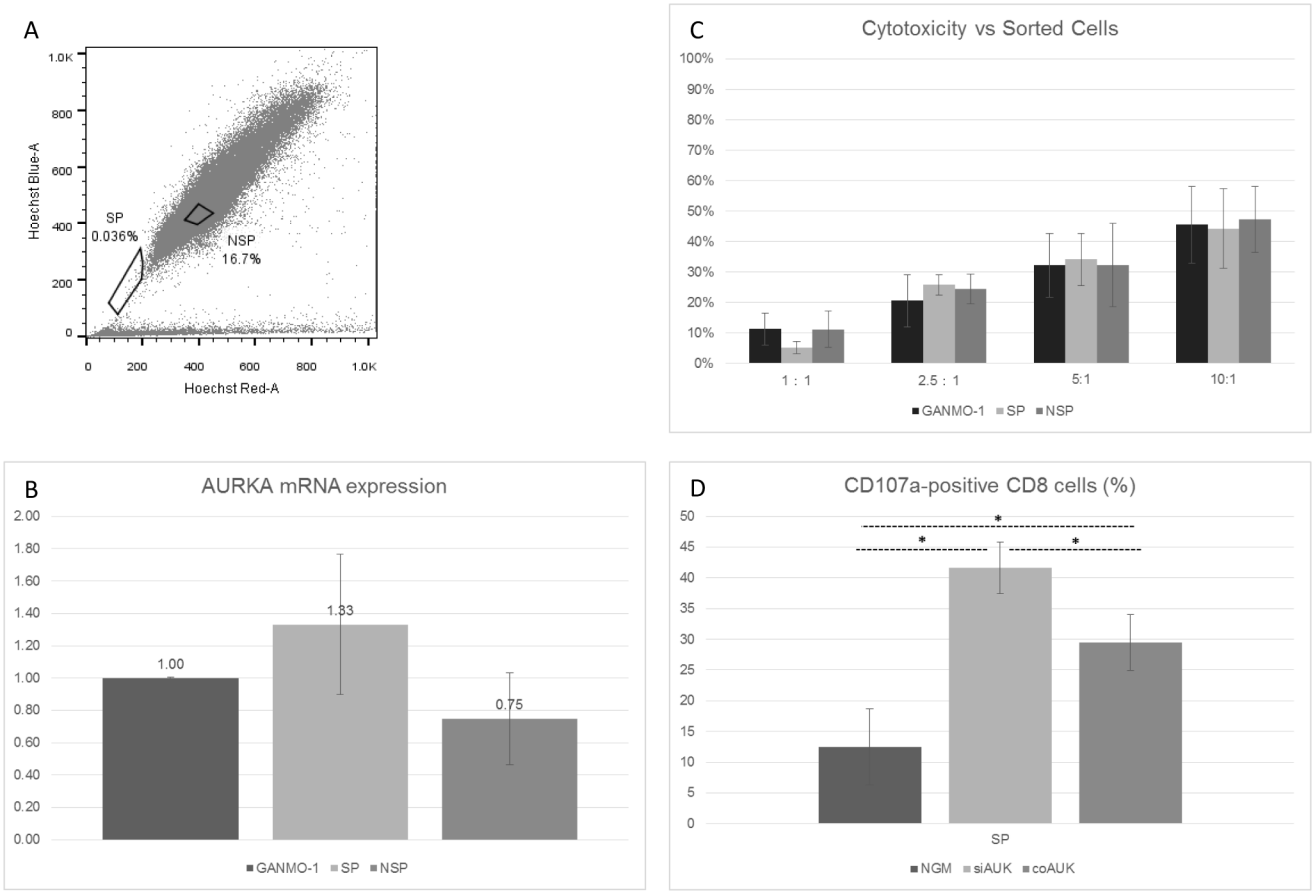
Characterisation of Side-Population (SP) cells, and Cytotoxic response against Side-Population cells. (A)—GANMO-1 cells were labelled with Hoechst 33342 and Propidium Iodide, and sorted by FACS. The ‘side population’ (SP) and a control ‘non-side population’ (NSP) gate are indicated. (B)–mRNA was collected from each sorted population, and unsorted GANMO-1 cells, and AURKA mRNA expression was determined by TaqMan RT-qPCR. (C)–Total cell killing was measured by Chromium-release assay following 3 hours co-culture of siAUK effector cells with unsorted GANMO-1 cells (‘Unsorted’), side-population cells, and non-side-population cells (n = 3). (D)–siAUK, coAUK, or NGM effector cells were co-cultured with SP cells for 3 hours in the presence of CD107a, then cells were analysed by FCM (* p<0.05, One-way ANOVA with Tukey’s test, n = 2, ± S.D.).

As putative cancer stem cells, ‘side population’ (SP) cells possess a number of stem cell-like characteristics, including high activity of ATP-binding cassette transporters [46]. This is the basis of the Hoechst 33342 dye-exclusion sorting utilised here (Fig 5A).

To verify ABC transporter function in GANMO-1 SP cells, we added 500μM Verapamil, an ABC transporter inhibitor, prior to Hoechst labelling. We found that preincubation with 500μM of Verapamil significantly reduced the population of SP cells (SP fraction of control = 0.04%, SP fraction with Verapamil added = 0.01%, p<0.05, paired t-test, n = 3. S5 Fig).

We also tested the capacity of SP cells to give rise to both SP and non-SP daughter cells. A reduced concentration of Hoechst 33342 (1μg/ml/10^6^ cells) was used for initial sorting. Despite the reduced dose, Hoechst-effluxing cells were still able to be distinguished from the bulk population (S6A Fig). An NSP was also sorted for comparison. Extended culture of SP and NSP progenitors up to day 12 (S6B Fig, NSP progenitors not shown) indicated that each was capable of generating both SP and non-SP daughter cell populations.

General progenitive capacity was also assessed by culturing of sorted SP and control cells in soft agar, such that mother and daughter cells would remain in physical proximity. After 12 days, an average of 68% of SP cells had evidently given rise to multiple daughter cells (average 4.58 cells per seed location) vs 28% for non-SP cells (average 1.67 per seed location), although these differences were non-significant (n = 3).

GANMO-1 SP cells were found to express low levels of CD33, CD38, and c-Kit (S7 Fig), giving them characteristics similar to human bone-marrow SP cells [47].

RNA was collected from the side-population cells, and AURKA mRNA levels were compared with those of unsorted GANMO-1 cells, and the control ‘non-side population’ (NSP) (Fig 5B). AURKA mRNA levels were comparable across the three populations, with those of the side-population cells tending to be higher, but not significantly so. This was consistent with Chromium release assays in which cytotoxicity mediated by siAUK cells was similar against each subpopulation (Fig 5C). We were also able to test the CD107a response by each effector cell type against SP cells, and it was clear that siAUK cells reacted more strongly than NGM or coAUK cells (Fig 5D).

CD34+ cord blood cells also express AURKA at detectable levels [4], and therefore might be targeted by AURKA-reactive CTLs. Elimination of these cells would impair normal haematopoiesis, and so we examined the CD107a response of siAUK cells against normal, CD34+ cord blood cells. The response by effector cells against HLA-A2+ cord blood cells was comparable to the response against the negative control, and HLA-A24+ cord blood cells (S8 Fig).

## Discussion

### Aurora Kinase A

The Aurora Kinase A (AURKA) protein has been identified as a suitable target for therapeutic strategies in a variety of cancer types as it plays a crucial role in the regulation of mitosis [2], and is overexpressed in a range of cancers [3, 4], and this expression correlates with accelerated disease progression, and a poor prognosis [6, 8]. AURKA plays a crucial role in cell division [2]. Whereas clonal evolution might allow certain cancers to evade immune surveillance by downregulation of antigenic genes (for example so-called ‘passenger mutations’), similar downregulation of AURKA expression in cancerous cells seems unlikely given its role as a driver of cancer progression.

While a number of conventional therapeutic candidates are under development [9, 10], there is as yet no reliable, universal strategy for treatment of AURKA-positive cancers. It is therefore an attractive candidate for immunotherapy, and a suitable TCR sequence for gene transfer to HLA-compatible patient T cells is available [4, 11].

Delivery of codon-optimised transgenic TCRs in conjunction with siRNA targeting the endogenous TCR sequences greatly increases the rate of formation of correctly-paired, transgenic TCRs [33–35], and the results to date strongly indicate that siTCR designs will be an essential component of clinical TCR transfer strategies. Nevertheless, in adapting verified TCRs to the siTCR system, it remains necessary to confirm that the efficacy of the original TCR is not lost in the context of the siTCR design.

### Expression of anti-AUK TCRs

The key advantage of the siTCR design is that it results in higher rates expression of correctly-paired TCR molecules, while decreasing the rate of mispairing between transgenic and endogenous α- and β-chains. The former is reflected here in the higher rates of binding of the target peptide, per copy of the siAUK vector, compared with each copy of the coAUK vector. We found that this did not result from differential rates of transgene expression from each vector, but was instead attributable to differences in vector design. Thus the siAUK design is superior with respect to binding of the AURKA peptide.

Direct quantitation of the binding of non-target peptides is not feasible. However the peptide-pulsed, cell killing assays do give an indirect measure of non-specific activity. Specifically, we quantitated cell killing of peptide-negative HLA-A*0201+ cells by non-gene modified (NGM), siAUK, and coAUK cells, and these results demonstrated reduced activity from the siAUK cells compared to the NGM cells, at most E:T ratios. There also appeared to be some reduction in non-specific activity from the coAUK cells also, which is consistent with high levels of expression of transgenic TCR chains [25]. However the lowest non-specific cytotoxic activity was from the siAUK cells, which was consistent with downregulation of endogenous TCR chains by the siTCR design [33, 34].

### Efficacy and sensitivity of the siAUK design

The response of siAUK and coAUK cells to peptide-pulsed HLA-A*0201 target cells was assessed across a range of parameters. The siAUK cells retained—and in some respects, exceeded—the responses shown by the coAUK cells. The data demonstrated that the siAUK cells were more sensitive (CD107a degranulation) to higher concentrations of the target peptide in the context of HLA-A*0201-positive target cells, yet the response at lower concentrations was not significantly different from the conventional vector. Furthermore, maximum cell killing (Chromium-release assays) was similar between siAUK cells and coAUK cells, although this level was achieved at a lower effector-cell ratio with siAUK cells. These results indicate that if utilising an AURKA specific TCR with the affinity of that used here in a clinical context, on-target off-cancer activity—targeting normal cells with low level expression of AURKA [7]–by siAUK cells would be no greater than by coAUK cells. At the same time, fewer transfused cells would be necessary to mount an effective response against cells with higher levels of AUKRA expression. This capacity is particularly relevant given the correlation between AUKRA expression levels and the aggressiveness and prognosis of AUKRA-positive cancers [5, 6]. Similarly, these results indicate that the non-specific response by siAUK cells would be lower than coAUK cells. All of these features are of course advantageous in a clinical context. These features may be logically attributed to the greater density of correctly-formed, and target-cognisant, TCRs on siAUK cells.

A similar array of experiments were also conducted with a series of HLA-A*0201+ cell lines with endogenous expression of AURKA. The response by the effector cells to endogenous expression of the target peptide were consistent with the results from the peptide-pulsing experiments, confirming the efficacy of the siAUK cells against AURKA peptide/HLA-A*0201 complex levels expressed by AURKA-positive leukaemias.

### Persistence of TCR Expression and Tetramer-binding by Gene-Modified Cells

Effective immunotherapy with gene-modified T cells requires the persistence and expansion of transfused cells *in vivo*. We confirmed the survival and proliferation of siAUK cells within a mixed cell population. Furthermore, the long-term culture results (Fig 4) indicated that the siAUK design gave a numerical advantage to the population of AURKA-reactive CD8+ cells, with a larger proportion of reactive cells in the starting population, compared with the coAUK design. This numerical advantage persisted for a number of weeks in expanding populations. In a clinical setting, the benefits of this numerical superiority are obvious. While our results indicate expansion of Tetramer-binding cells within the coAUK Vβ12+ population, it must be remembered that such cells retain their endogenous TCR chains, and will therefore still present mispaired TCRs. At this time, there is no post-hoc method available to specifically eliminate cells bearing mispaired TCRs from the effector cell population. Finally, the evidence of exhaustion within both effector cell populations highlights the importance of selecting the correct populations of T cells for transduction and transfusion [48].

### Reactivity Against Side-Population cells

While the definition, and even the existence, of cancer stem cells is disputed by some, their putative role in the survival and proliferation of cancers makes them an attractive target [46]. Cancer stem cells share various characteristics with normal stem cells, including the activity of ATP-binding cassette transporters, which are also responsible for drug efflux from such cells [37]. As there is no universal cell-surface marker for stem cells, the identification and isolation of Hoechst-effluxing cells, the ‘side population’ (SP), has become a *de facto* model for cells with stem-like characteristics [36, 49, 50].

In the context of cancer stem cells, drug efflux can impair tumour response to chemotherapies [38]). As immunotherapies are not dependent upon drug uptake by target cells, they may hold some potential in attacking cancer stem cells. Accordingly, we examined that activity of gene-modified T cells against a model for cancer stem cells.

Our results demonstrated cytotoxicity against GANMO-1 SP cells, at levels comparable to unsorted and control populations. By contrast, there was no response against normal cord blood cells, likely due to lower AURKA expression in these cells [4] combined with lower HLA expression in less-differentiated cell types [51]. Both factors are likely to reduce targeting by AURKA-reactive, HLA-A*0201-specific CTLs. Therefore the strategy of cancer immunotherapy appears to have some potential against cancer stem cells, and thus is worthy of further investigation.

### Conclusions

TCRs and Chimaeric Antigen Receptors (CARs) are both valuable tools for cancer immuno-gene therapy, each with their own strengths and weaknesses [13, 52–54]. The use of TCRs in gene-transfer immunotherapy is complicated by the presence of existing TCR genes in many of the best candidate effector cell types [16], which leads to a range of complications (as outlined in the introduction).

The siTCR vector design has been shown to address many of these factors [33, 34], and consequently the aim of this study was to test and confirm the efficacy of the siTCR vector design when applied to anti-AURKA TCR α- and β-chains. It was expected that the siTCR design would result in higher rates of correctly-formed, AURKA cognisant TCRs, per vector copy number [33, 34]. We showed this to be true.

The features of this design confer numerous advantages to TCR cancer immuno-gene therapy strategies.

The higher rate of TCR presentation per vector copy number means that lower titres of vector can be used, whatever the backbone. This has direct benefits with respect to genotoxic risk [26, 27]. Higher actual and effective titres also mean that a greater number of effector cells can be transfused, with less *ex vivo* expansion required. This saves time, but also means that the transfused population is younger, which has been associated with anti-tumour efficacy [55]. Finally, as mentioned above, the reduction in mispaired TCRs has the direct benefit of reducing cross-reactivity against non-target proteins.

In conclusion, we have been able to confirm the benefits and adaptability of the siTCR concept, and in so doing strengthened the conclusion that such a design is an essential component of TCR gene transfer strategies. We also established the superiority and efficacy of this design in the context of the AUKRA tumour-associated antigen, to such an extent that this vector design might be considered ready to move on to clinical trials.

## Supporting Information

S1 Fig Sketch of ‘siAUK’ and ‘coAUK’ retroviral vectors. The α- and β-chains of the AURKA-specific TCR were linked with a self-cleaving 2A peptide. The siAUK vector also included an siRNA cluster (A). The coAUK vector lacked the siRNA cluster, but was otherwise identical (B). All of these elements were expressed from the 5’ LTR promoter.
(TIF)

S2 Fig Vβ12 expression by CD8+ effector cells, relative to vector copy number. CD8+ cells from three donors were transduced with siAUK or coAUK vectors, at a range of MOIs. Expression of the Vβ12 chain of the transgenic TCR was analysed by flow cytometry. Genomic DNA was also collected from the cells, with vector copy number determined by qPCR. Correlation coefficients were compared using Preacher’s calculation (p>0.05).
(TIF)

S3 Fig Binding of AURKA A207 tetramer by CD8+ effector cells, relative to Vβ12 expression. CD8+ cells from three donors were transduced with siAUK or coAUK vectors, at a range of MOIs. Binding of fluorophore-labelled A207 tetramer was analysed by flow cytometry. Cells were also labelled with an antibody to the Vβ chain of the transgenic TCR. Genomic DNA was also collected from the cells, with vector copy number determined by qPCR. Correlation coefficients were compared using Preacher’s calculation (p<0.05).
(TIF)

S4 Fig AURKA mRNA expression in GANMO-1 cells. Total RNA was harvested from GANMO-1 cells, and expression of AURKA mRNA (relative to GapDH housekeeping gene) was normalised to K562 cells, with PBMC also included for comparison.
(TIF)

S5 Fig Inhibition of side-population cell ABC transporters. GANMO-1 cells were preincubated with Verapamil before labelling. The SP gate was based on the controls (A). Addition of Verapamil lead to a significant diminution of the SP cells (B). These plots are representative of three trials.
(TIF)

S6 Fig Progenitive capacity of side-population cells. For assays involving extended culture of SP cells, GANMO-1 cells were labelled with Hoechst 33342 at 1μg/ml/10^6^ cells, sorted into SP and NSP populations (A). SP cells were cultured for 12 days in 96-well, round-bottomed plates. Cells were re-labelled, with Hoechst 33342 at 5μg/ml/10^6^ cells, then analysed by flow cytometry. The initial SP cell population gave rise to both SP and non-SP cell types (B).
(TIF)

S7 Fig Cell-surface labelling of side-population cells. GANMO-1 cells were labelled with Hoechst 33342, and a range of cell surface markers, CD33 (A), CD38 (B), and c-Kit (C), then analysed by flow cytometry.
(TIF)

S8 Fig CD107a degranulation response by siAUK effector cells co-cultured with normal CD34+ cord blood cells. Cord blood from HLA-A2 and HLA-A24 donors were co-cultured with siAUK effector cells in the presence of CD107a antibody, under standard conditions. Peptide-pulsed and un-pulsed C1R-A2 cells were used as positive and negative controls.
(TIF)
