# Supplementary figures and images for: A Functionally Superior Second-Generation Vector Expressing an Aurora Kinase-A-Specific T-Cell Receptor for Anti-Leukaemia Adoptive Immunotherapy
Source: PLoS One. 2016 Jun 7;11(6):e0156896. doi: 10.1371/journal.pone.0156896 (PMC4896450; doi:10.1371/journal.pone.0156896)

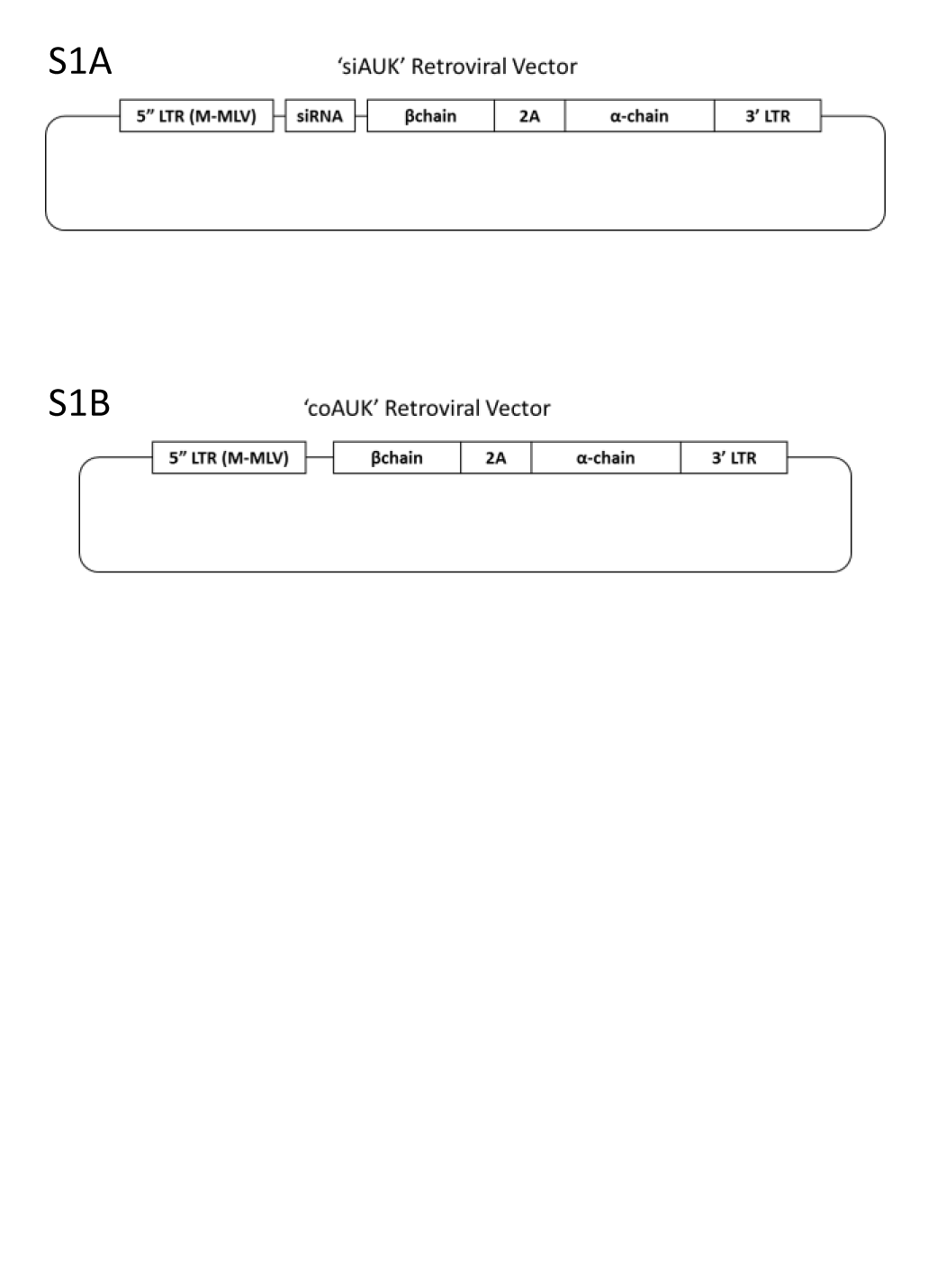

Supplement: S1 Fig — The α- and β-chains of the AURKA-specific TCR were linked with a self-cleaving 2A peptide. The siAUK vector also included an siRNA cluster (A). The coAUK vector lacked the siRNA cluster, but was otherwise identical (B). All of these elements were expressed from the 5’ LTR promoter. (TIF) [file pone.0156896.s001.tif]

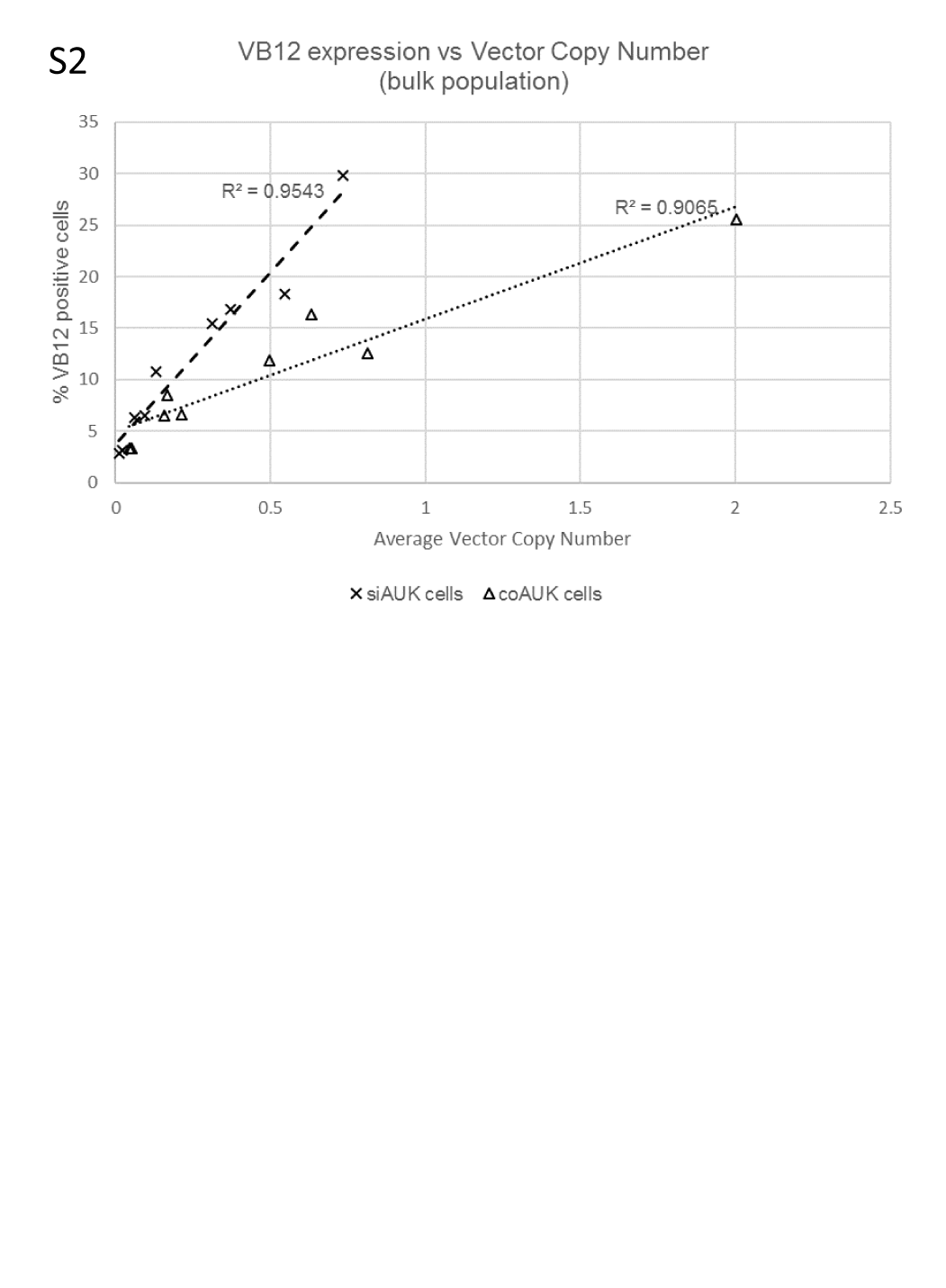

Supplement: S2 Fig — CD8+ cells from three donors were transduced with siAUK or coAUK vectors, at a range of MOIs. Expression of the Vβ12 chain of the transgenic TCR was analysed by flow cytometry. Genomic DNA was also collected from the cells, with vector copy number determined by qPCR. Correlation coefficients were compared using Preacher’s calculation (p>0.05). (TIF) [file pone.0156896.s002.tif]

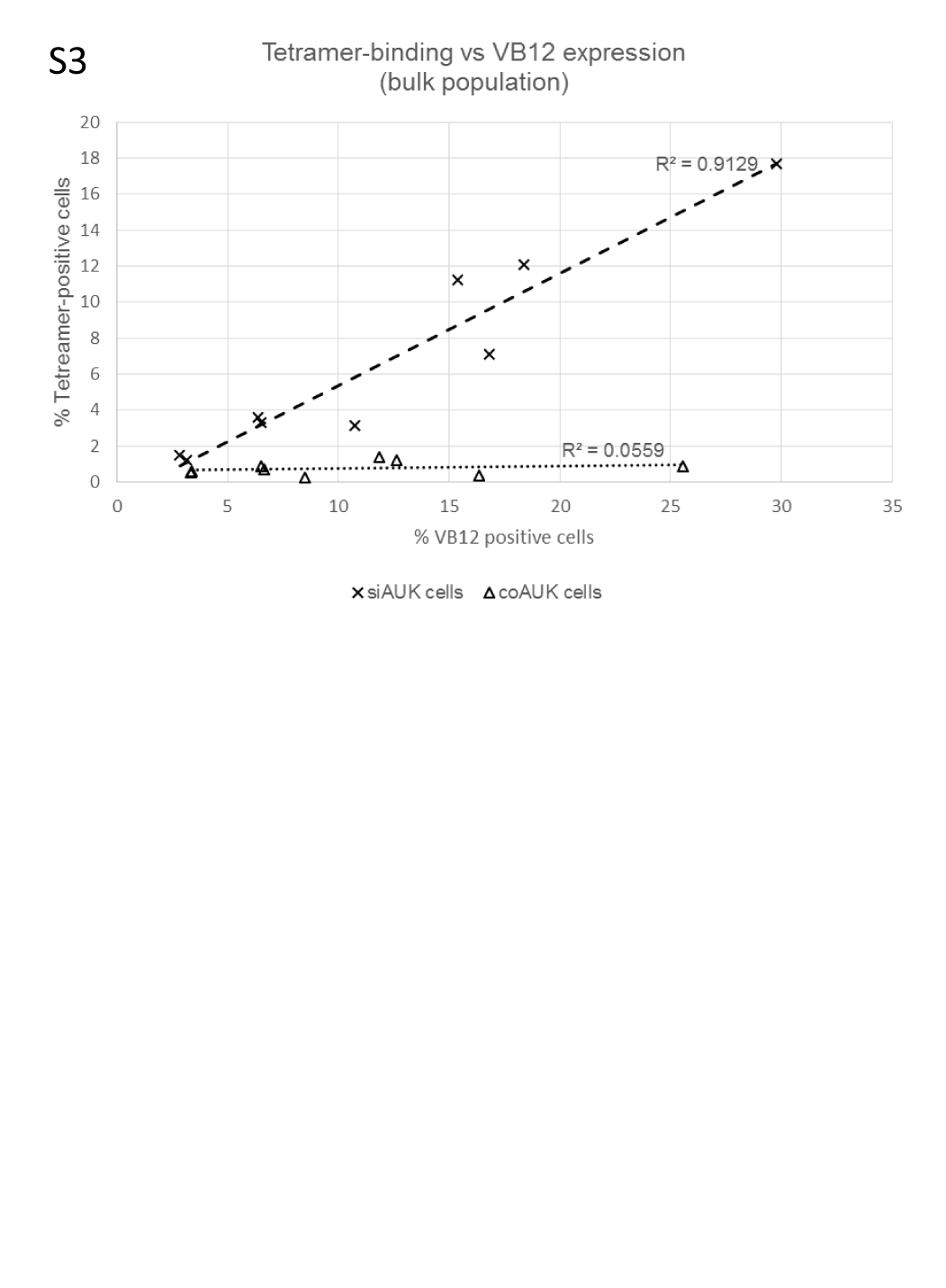

Supplement: S3 Fig — CD8+ cells from three donors were transduced with siAUK or coAUK vectors, at a range of MOIs. Binding of fluorophore-labelled A207 tetramer was analysed by flow cytometry. Cells were also labelled with an antibody to the Vβ chain of the transgenic TCR. Genomic DNA was also collected from the cells, with vector copy number determined by qPCR. Correlation coefficients were compared using Preacher’s calculation (p<0.05). (TIF) [file pone.0156896.s003.tif]

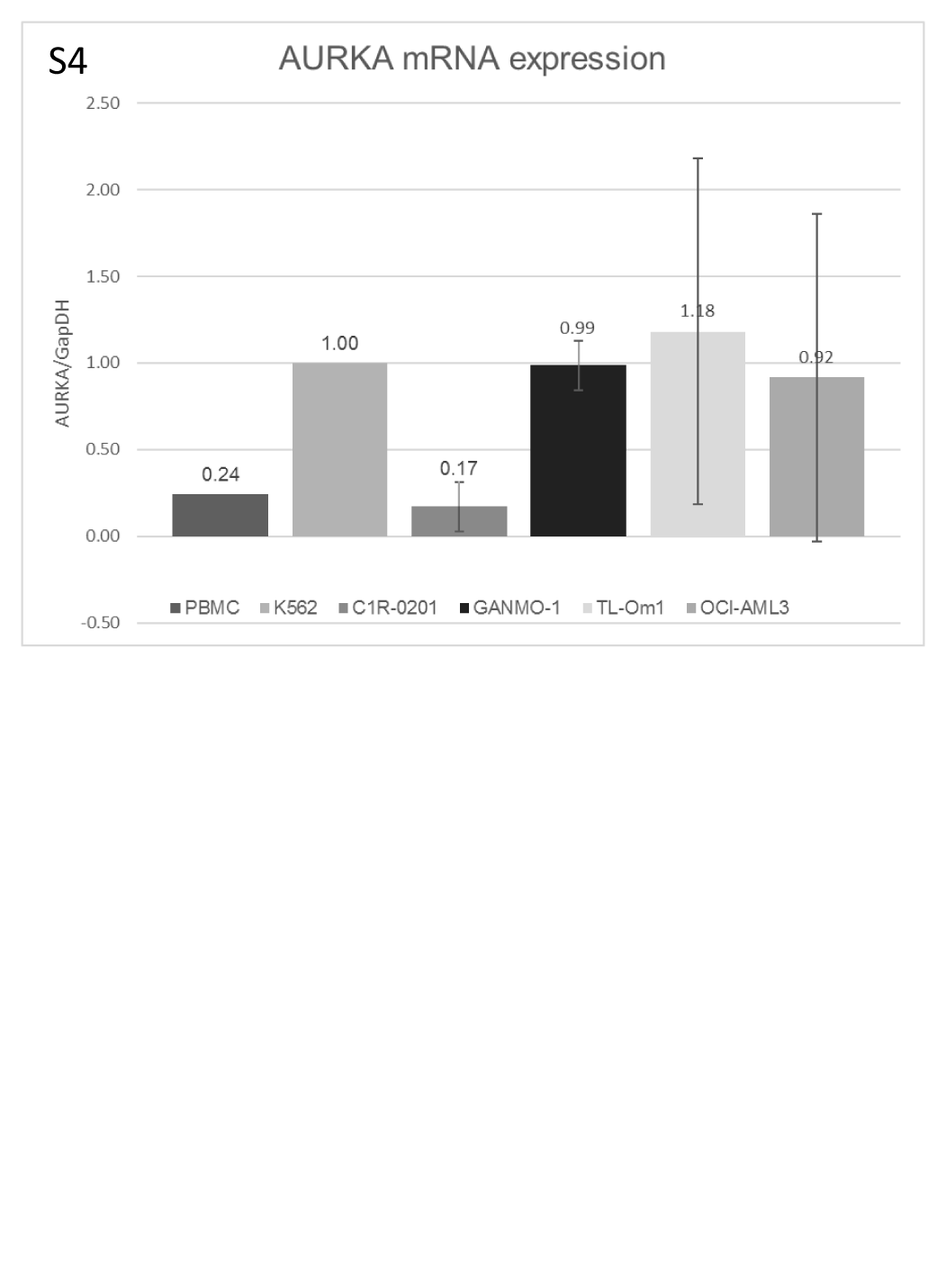

Supplement: S4 Fig — Total RNA was harvested from GANMO-1 cells, and expression of AURKA mRNA (relative to GapDH housekeeping gene) was normalised to K562 cells, with PBMC also included for comparison. (TIF) [file pone.0156896.s004.tif]

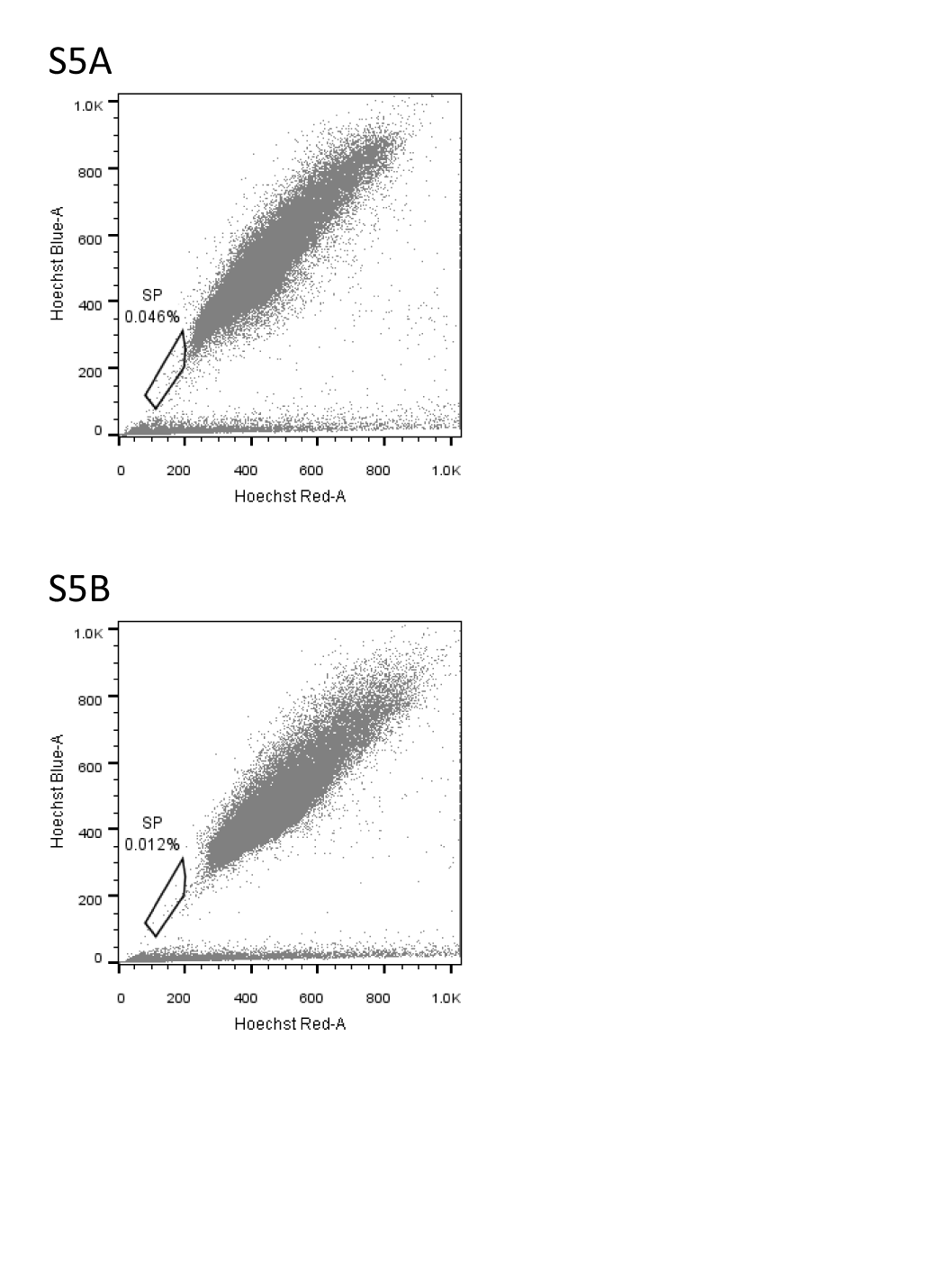

Supplement: S5 Fig — GANMO-1 cells were preincubated with Verapamil before labelling. The SP gate was based on the controls (A). Addition of Verapamil lead to a significant diminution of the SP cells (B). These plots are representative of three trials. (TIF) [file pone.0156896.s005.tif]

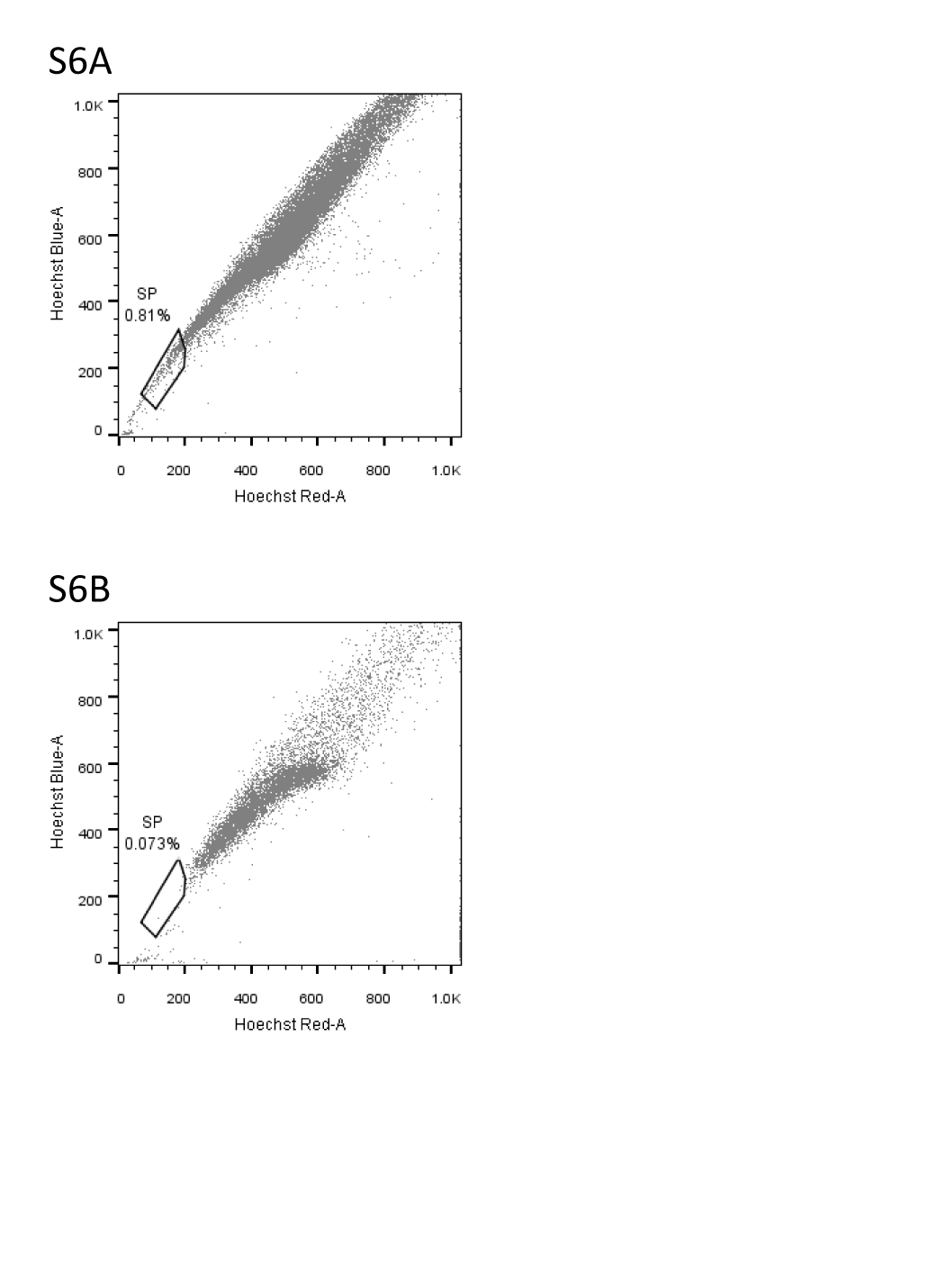

Supplement: S6 Fig — For assays involving extended culture of SP cells, GANMO-1 cells were labelled with Hoechst 33342 at 1μg/ml/106 cells, sorted into SP and NSP populations (A). SP cells were cultured for 12 days in 96-well, round-bottomed plates. Cells were re-labelled, with Hoechst 33342 at 5μg/ml/106 cells, then analysed by flow cytometry. The initial SP cell population gave rise to both SP and non-SP cell types (B). (TIF) [file pone.0156896.s006.tif]

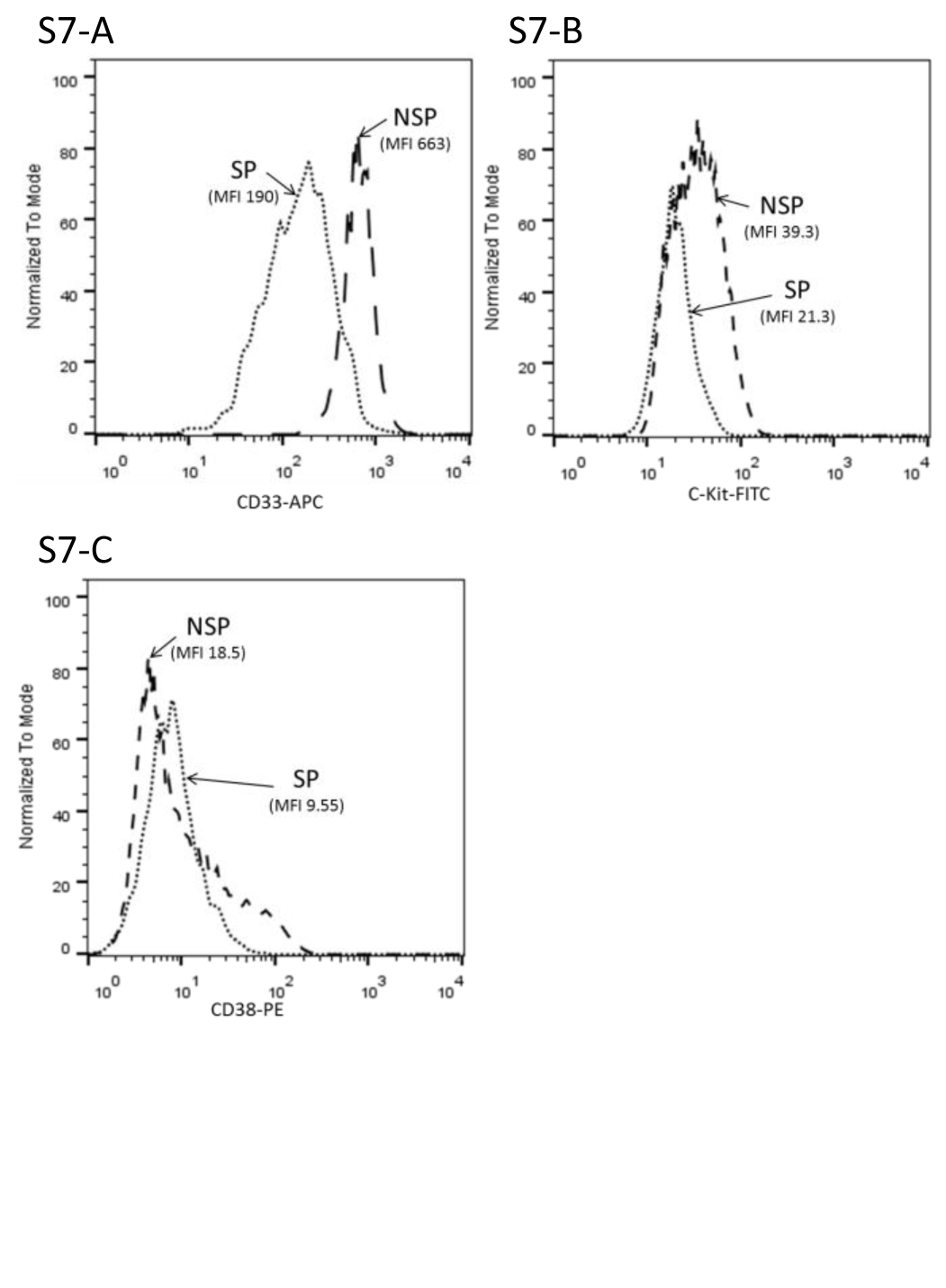

Supplement: S7 Fig — GANMO-1 cells were labelled with Hoechst 33342, and a range of cell surface markers, CD33 (A), CD38 (B), and c-Kit (C), then analysed by flow cytometry. (TIF) [file pone.0156896.s007.tif]

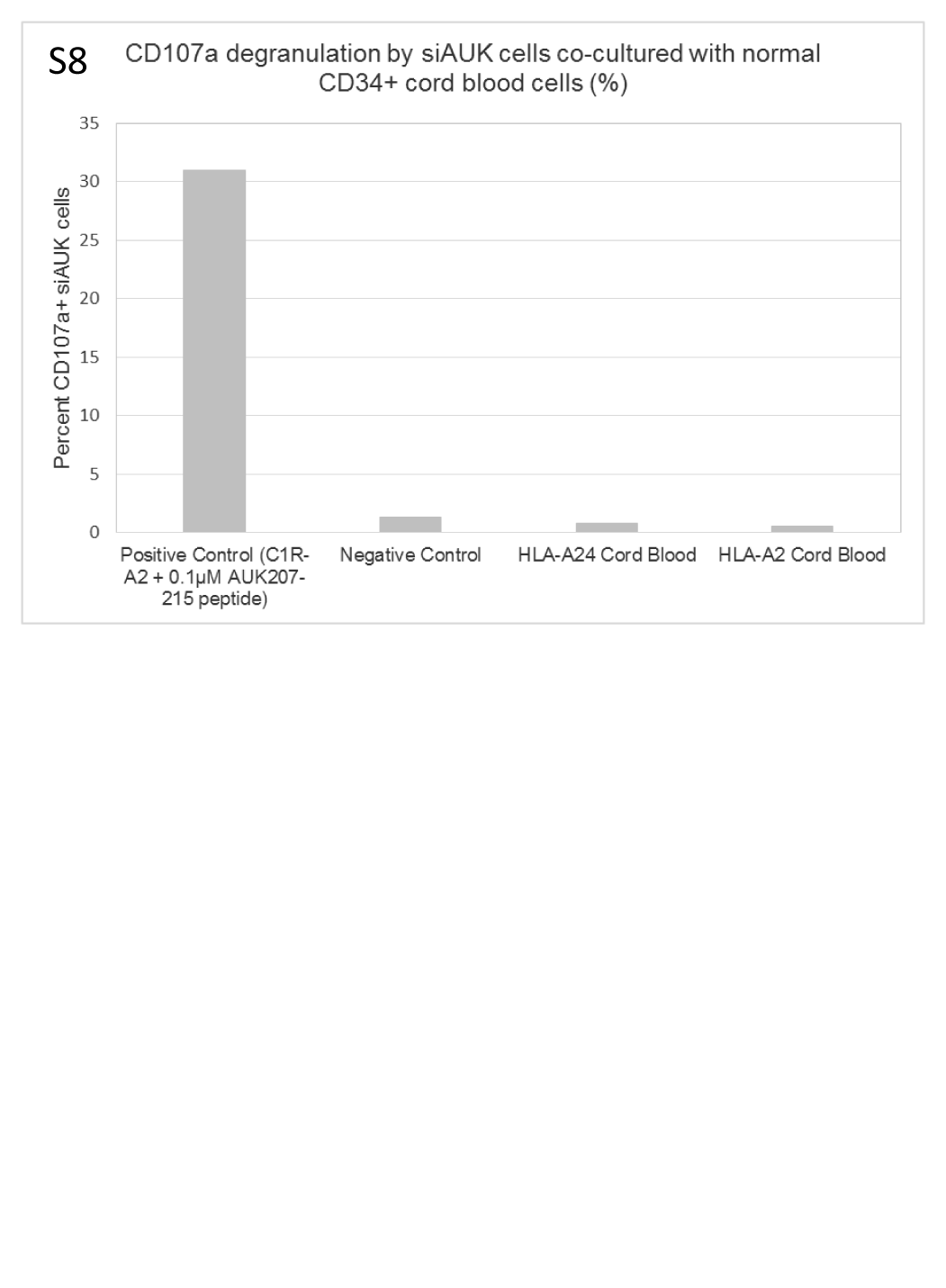

Supplement: S8 Fig — Cord blood from HLA-A2 and HLA-A24 donors were co-cultured with siAUK effector cells in the presence of CD107a antibody, under standard conditions. Peptide-pulsed and un-pulsed C1R-A2 cells were used as positive and negative controls. (TIF) [file pone.0156896.s008.tif]
